# Integrating phase-rectified signal averaging with machine learning to predict stroke-associated infections: a retrospective cohort study

**DOI:** 10.3389/fneur.2025.1653947

**Published:** 2026-01-13

**Authors:** Yiyang Gao, Jiaqi Zhong, Tingting Li, Chuanbin Yang, Jiajun Yang

**Affiliations:** 1 Department of Neurology, Shanghai Sixth People's Hospital Affiliated to Shanghai Jiao Tong University School of Medicine, Shanghai, China; 2 Shanghai Neurological Rare Disease Biobank and Precision Diagnostic Technical Service Platform, Shanghai, China; 3 Neurological Disorder Center, Haikou Orthopedic and Diabetes Hospital of Shanghai Sixth People's Hospital, Haikou, China

**Keywords:** acute ischemic stroke, machine learning, phase-rectified signal averaging, prediction, stroke-associated infection

## Abstract

**Background:**

Stroke-associated infection (SAI) adversely affects the prognosis of acute ischemic stroke (AIS) patients, contributing to poorer functional outcomes and survival. The absence of validated tools for early SAI diagnosis and risk stratification in AIS remains a critical clinical gap. This study aims to develop and validate a machine learning-based prediction model that leverages phase-rectified signal averaging (PRSA) indicators closely linked to SAI pathogenesis for timely risk assessment in emergency settings.

**Methods:**

This derivative cohort comprised 392 patients diagnosed with AIS between 2021 and 2023. The variables considered in this study included age, sex, heart rate variability (HRV) parameters, and PRSA parameters. Variable selection was performed using the Boruta algorithm and correlation analysis. Ten machine learning methods were employed to construct the SAI diagnostic model, and its performance was evaluated using the area under the curve (AUC), decision curve analysis (DCA), sensitivity, specificity, and an internal validation cohort. The predictive model outcomes were interpreted using Shapley Additive Explanations (SHAP).

**Results:**

Through variable screening, 16 indicators were identified as independent predictive factors for SAI in AIS patients. Utilizing these indicators, 10 machine learning models were developed. Among the machine learning algorithms, the Categorical Boosting (CAT) model demonstrated superior performance, achieving an accuracy of 91%, sensitivity of 88%, specificity of 92%, F1-score of 74%, and an AUC of 0.939 (95% CI: 0.894–0.984). Furthermore, SHAP identified cardiac deceleration capacity (DC) and the National Institute of Health Stroke Scale (NIHSS) at admission as the primary determinants influencing the predictions of the machine learning models.

**Conclusion:**

Machine learning algorithms, when integrated with demographic and clinical factors, demonstrated accurate prediction of SAI in patients with AIS. The CAT model exhibited robust performance, highlighting its potential to enhance early detection and treatment in clinical practice. Additionally, PRSA markers may serve as potential targets for preventive interventions, enabling more judicious, timely, and targeted use of antibiotics. This approach opens new avenues for research into the prophylactic management of SAI.

## 1 Introduction

Stroke is the most common and serious manifestation of cerebrovascular disease and is the leading cause of hospitalization for neurological disorders (1). Ischemic stroke accounts for the majority of cerebrovascular disease (2), and acute ischemic stroke (AIS) is a condition in which localized ischemia is caused by the narrowing or occlusion of the lumen of a blood vessel due to embolism, severe hypoperfusion, and thrombosis (3). Stroke-associated infections (SAIs) are a common and serious complication of stroke, including stroke-associated pneumonia (SAP), urinary tract infections (UTIs), and other infections diagnosed within the first week of stroke, which occur in 5–65% of patients (4, 5). Early studies have shown that SAIs are associated with increased mortality and prolonged hospital stays compared to uninfected patients (6, 7). They lead to the need for long-term rehabilitation and care, which can increase family burden and healthcare costs.

Conventional indicators of inflammation (WBC, CRP, PCT, etc.) have an important role in determining the presence or absence of post-stroke infections, but most of them are serological indicators that are not easy to obtain and monitor the dynamics of infection on a daily basis. Currently, several studies have found that many indicators related to inflammation and stress may be helpful in predicting the occurrence of SAI, but they are not routine indicators and are not conducive to large-scale clinical dissemination. This means that there is a greater need for more sensitive, accessible, and multidimensional markers of SAI to aid in early detection (8).

SAI is associated with post-stroke stress and immunosuppression. Severe autonomic nervous system deficits, dysregulation of the balance between sympathetic and parasympathetic activity, are also relevant risk factors for SAI. Some studies have reported significant autonomic nervous system dysfunction in 76% of AIS patients, mainly manifested by activation of the sympathetic nervous system (9); a rapid increase of norepinephrine in serum was observed in rats after middle cerebral artery occlusion (10); sympathetic hyperactivity and an increase in norepinephrine can lead to stroke-induced immunosuppression, making patients susceptible to infections (11). Therefore, sympathetic hyperactivity is associated with the development of SAI. It has been found that heart rate variability (HRV) indices and phase-corrected signal averaging (12) reflect these changes in the autonomic nervous system in stroke patients. Phase-rectified signal averaging (PRSA) is a signal processing technique developed to assess autonomic function by quantifying heart rate acceleration capacity (AC) and deceleration capacity (DC). Initially, it was proposed that its derived indices, AC and DC, could separately reflect sympathetic and vagal nervous activity. However, this direct physiological mapping has been questioned (13). Subsequent theoretical work by Rivolta et al. demonstrated that for stationary signals, AC and DC are equal in magnitude but opposite in sign, suggesting they primarily reflect the overall capacity for heart rate changes rather than distinct autonomic branches in steady-state conditions. The difference between DC and AC, termed deceleration reserve (DR), has thus been introduced as a potentially more sensitive marker for autonomic imbalance, particularly under non-stationary conditions, such as those induced by pathological stress (13). In this study, we used this technique to investigate the autonomic function of patients with AIS (14).

In this retrospective study, we aimed to develop a model for the early diagnosis of SAI by using AC, DC, and HRV parameters in conjunction with common clinical serological samples. The modeling approach used 10 machine learning methods, namely Logistic Regression (LR), Random Forest (RF), Support Vector Machine (SVM), Extreme Gradient Boosting (XGB), Gradient Boosting Machine (GBM), K-Nearest Neighbors (KNN), Adaptive Boosting (ADA), Light Gradient Boosting Machine (LGBM), Neural Network (NNET), and Categorical Boosting (CAT) to construct the model. The efficacy of the various algorithmic models was compared through internal validation, and their predictive power was assessed to identify the optimal model. So far, however, there have been a lack of studies to explore the correlation between AC, DC, and HRV parameters with SAI. In this study, we aim to uncover this relationship.

## 2 Methods

### 2.1 Research design

**Figure 1:**
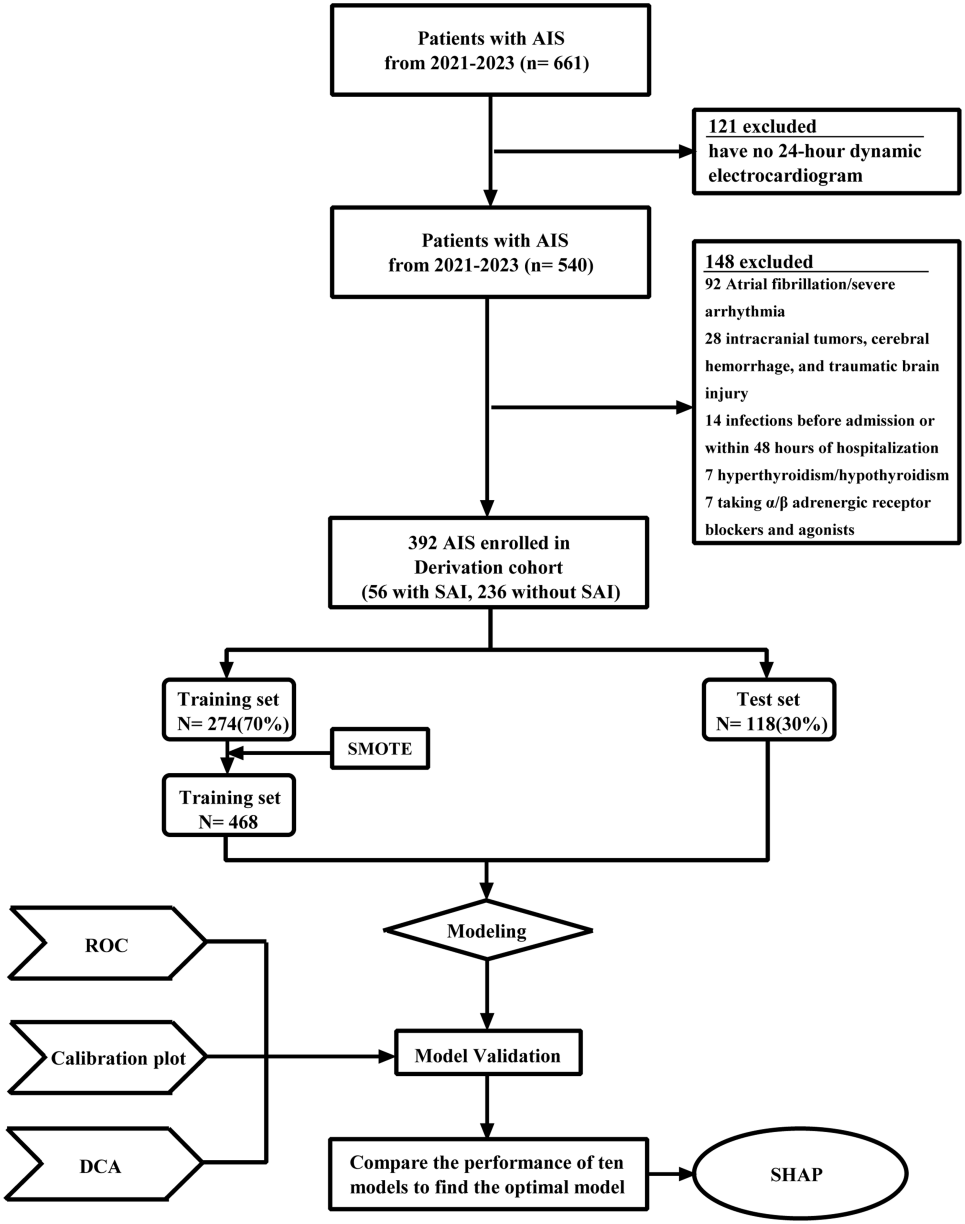
Flowchart of patient selection and machine learning model development process. AIS, acute ischemic stroke; SAI, stroke-associated infection; ROC, receiver operating characteristic curve; SMOTE, synthetic minority oversampling technique; DCA, decision curve analysis; SHAP, shape additive explanation.

The research design for this study is depicted in Figure 1 and comprises of three steps: development, internal validation, and interpretation. Initially, a training cohort, constituting 70% of the derivation cohort, was used to develop predictive models. Subsequently, the remaining 30% of the derivation cohort was designed for internal validation. The dataset employs a 70/30 split stratified by subject ID, strictly preventing the same patient data from appearing simultaneously in both the training and test sets, thereby eliminating the risk of data leakage. We assessed average machine learning model performance by calculating the area under the receiver–operator-characteristics (AUC), sensitivity, and specificity. The Shapley Additive explanations (SHAP) algorithm was utilized to elucidate the significance of features in the predictive model and to identify non-linear relationships among risk predictors.

### 2.2 Study subjects

Inclusion criteria were as follows: (a) Patients meeting the diagnostic criteria of the 2018 Chinese Guidelines for the Diagnosis and Treatment of Acute Ischemic Stroke; (b) Patients aged 18 years or older; (c) Patients whose onset time was less than or equal to 24 h; (d) Patients who underwent a 24-h dynamic electrocardiogram during hospitalization; (e) Patients who signed an informed consent form.

The exclusion criteria were as follows: (a) Patients with a history of intracranial tumors, intracranial infections, cerebral infarction within 1 year, and other intracranial lesions; (b) Patients with a history of severe arrhythmia (atrial fibrillation, frequent premature beats, atrioventricular block of more than second degree), heart failure; (c) Patients with endocrine diseases affecting autonomic nervous function; (d) Patients who had used medications affecting autonomic nervous function, such as *α*/*β* adrenergic receptor blockers and agonists; (e) Patients with a history of infection within 2 weeks prior to stroke onset; (f) Patients who take antibiotics, steroids, immunosuppressive drugs, etc. prior to admission. Patients were divided into SAI and NSAI groups according to the occurrence of stroke-associated infections within 3–7 days after admission. SAI was diagnosed by the treating physician based on clinical symptoms, and/or suggestive clinical examination, and/or radiological findings, and/or microbiological evidence of infection.

The derivation cohort included patients treated at our hospital from June 2021 to August 2023, diagnosed with AIS. A total of 661 individuals meeting these criteria were screened for participation.

Based on the exclusion criteria, 121 patients were not included. 92 patients had been diagnosed with atrial fibrillation or severe arrhythmia, while 28 patients were not included in this study due to their past or current history of intracranial tumors, cerebral hemorrhage, and traumatic brain injury. Additionally, 14 patients developed infections before admission or within 48 h of hospitalization. In addition, seven patients with a history of hyperthyroidism or hypothyroidism and seven patients taking *α*/*β* adrenergic receptor blockers and agonists were excluded from this study. Consequently, a total of 392 eligible patients were selected for the derivation cohort. Among these patients, 56 were diagnosed with post-stroke infection, forming the stroke-associated infection group. The remaining 236 patients showed no signs of infection, constituting the non-stroke-associated infection group.

### 2.3 Data collection

This study collected patient data on demographic information (gender, age, height, weight, and body mass index), past and personal history (History of hypertension, diabetes, smoking, and drinking), treatment information (nasogastric tube, urinary catheterization, and thrombolysis), and laboratory test results of infection indicators by reviewing electronic medical records and the laboratory management system. The laboratory test results included various aspects such as features of infection (CRP, WBC, NLR, SIRI, N, etc.), tumor markers (CEA, AFP, CA125, CA199, etc.), and hormones related to the autonomic nervous system (FT3, FT4, and TSH). Furthermore, we included the National Institute of Health Stroke Scale (NIHSS) at admission, and the presence of hemorrhagic transformation as additional features, along with laboratory values. The full names and abbreviations of the included features are listed in Supplementary Table S1.

### 2.4 Collection and significance of AC, DC, and classical HRV parameters

All study subjects underwent 24-h dynamic electrocardiogram (Holter ECG) monitoring within 48 h of admission, with a standardized recording period from 8:00 a.m. on the day of admission to 8:00 a.m. the following day. Records longer than 24 h were truncated, while shorter records were excluded from analysis. This ensured that all features were computed from comparable time intervals. Each feature was automatically analyzed using the Cardioscan-12 software, and each patient received a unique, representative scalar value, which reflects the overall condition throughout the entire analysis period. PRSA analysis was performed to derive AC and DC, employing the technique described by Bauer et al. (15), with parameters set to T (anchor point definition) = 1, s (quantification scale) = 2, and L (window length) = 50. This configuration is implemented by default in the Cardioscan-12 software, ensuring consistency with prior cardiovascular studies (15).

The PRSA technique quantifies the overall propensity for AC or DC. While decreases in DC have been empirically linked to poorer clinical outcomes in various conditions, interpreting DC and AC as direct and exclusive measures of vagal and sympathetic activity, respectively, is an oversimplification (13). The thresholds for DC (e.g., >4.5 ms, 2.6–4.5 ms, ≤2.5 ms) and extreme values of AC and DC should be viewed as risk-stratification tools rather than precise indicators of vagal “tone.” In line with recent research highlighting the importance of autonomic imbalance (13), we calculated the DR, defined as DR = DC + AC. This metric, theoretically zero for a stationary Gaussian process, becomes positive when deceleration capacity predominates and negative when acceleration trends dominate, potentially offering a robust measure of the net autonomic state under the non-stationary conditions following stroke (13).

The HRV parameters include the standard deviation of normal R–R intervals (SDNN), the standard deviation of average normal to normal R–R intervals (SDANN) every 5 mines, the root mean square of successful R–R difference (RMSSD), the percentage of successful normal sinus R–R intervals with absolute changes exceeding 50 ms (PNN50), low-frequency power (LF), high-frequency power (HF), very low frequency (VLF), and the ratio of LH to HF (LF/HF). According to the former study, it found that LF is an index of both sympathetic and parasympathetic activity, and HF represents the most efferent vagal (parasympathetic) activity to the sinus node (16). VLF partially reflects thermoregulatory mechanisms, fluctuation in activity of the renin–angiotensin system, and the function of peripheral chemoreceptors. The LF/HF ratio stands for the sympathovagal balance (16). The RMSSD and PNN50 are associated with HF and hence parasympathetic activity, whereas SDNN is correlated with LF and reflects the overall activity of the autonomic nervous system (17).

### 2.5 Statistical analysis

Due to the retrospective nature of this study, some clinical information is lacking. Variables with more than 20% missing data were excluded from the analysis, while those with fewer than 20% missing data were imputed via the missRanger package to ensure unbiased estimates. The missRanger package is based on the random forest algorithm (18). The specifics of the missing values are shown in Supplementary Figure S1.

Baseline data analysis of patients began with normality tests on the quantitative data. Normally distributed continuous data were presented as mean ± SD, and comparisons between groups were conducted using independent samples *t*-tests. Skewed data were described using the median (P25, P75), with group comparisons performed via the Mann–Whitney U tests. Count data were expressed as frequency (percentage, %), with chi-squared tests used for statistical analysis.

### 2.6 Variable selection

Boruta’s algorithm, an extension of the RF algorithm, identifies key variables by comparing the *Z* value of each true feature with that of corresponding “shadow features.” Features with significantly higher *Z* values than shadow features were deemed “important” (green area), while those without significant differences were marked as “unimportant” (red area) (19). Boruta’s algorithm analysis was then used to finalize the variables for inclusion, thereby eliminating any redundant features.

Although the Boruta algorithm can effectively screen out features with high predictive value, they do not guarantee that the selected feature set is mutually independent. To address the potential issue of high correlation among features and avoid the adverse effects of multicollinearity on model stability and interpretability, we introduced an additional correlation analysis step after obtaining the common feature subset from Boruta.

We computed the Spearman rank correlation coefficients between all variables in this feature subset. This non-parametric correlation test imposes no assumptions on variable distributions and captures non-linear relationships. We established an empirical correlation threshold of |*ρ*| > 0.8. When the correlation between a pair of features exceeded this threshold, we discarded the feature with lower average importance in Boruta algorithms, retaining the other feature with higher importance. This ensured minimal redundancy within the final feature set.

Ultimately, we obtained a final feature set that is both highly predictive and significant, while maintaining relative independence among features. This set will be utilized for subsequent model training and interpretation.

### 2.7 Model derivation and validation

The machine learning algorithm models were developed using R version 4.4.1. The dataset was divided into training and test sets in a 7:3 ratio. The Synthetic Minority Oversampling Technique (SMOTE) is an efficient algorithm for solving the class imbalance problem, which employs K-neighborhood synthesis to focus on a finite number of classes to obtain a balanced dataset (20). In the R’s themis package, K defaults to 5, which is suitable for many common scenarios based on empirical background. Therefore, we use SMOTE to address the data imbalance and reduce model overfitting. SMOTE is only applied to our training set, and we do not oversample the test set, thus maintaining the natural frequency of the results. The variable features of the training set after applying SMOTE and the test sets are shown in Supplementary Tables S2, S3, respectively. Various machine learning algorithms were applied, including GBM, RF, LR, SVM, KNN, NNET, CAT, ADA, LGBM, and XGB. The machine learning classifiers used in this study take as input a fixed-dimensional feature vector. Each patient is represented by a vector composed of all their feature values. Therefore, the entire dataset forms a matrix of dimensions (N patients × M features), which is directly utilized for model training and testing. Hyperparameter tuning was conducted using grid search and an internal 10-fold cross-validation procedure to optimize model performance. After selecting the optimal hyperparameters, the model was retrained on the complete training subset to finalize the weighting and generate a locked model. These locked models were then assessed on the internal validation cohort. The performance of each model was evaluated using Receiver Operating Characteristic curves (ROC) and corresponding AUC values. Clinical usefulness was assessed using decision curve analysis (DCA), and calibration curves were generated to evaluate the accuracy of risk predictions. Ultimately, the most optimal model was selected for SHAP further.

## 3 Result

### 3.1 Patient characteristics

This study conducted an initial comparison between two study groups: the SAI group (56 individuals) and the no SAI group (336 patients). A comparison of baseline characteristics between the two groups is shown in Table 1. We used the SMOTE algorithm on the training set for data imbalance. The original training set of 274 cases contained 39 SAI cases, 235 no SAI cases, with 14.49% of SAI cases. There is a serious imbalance. After resampling the training set, the processed data of 468 cases contained 234 no SAI cases, 234 SAI cases, with 50.00% of SAI cases.

**Table 1:** Baseline characteristics of each indicator.

Variables	Total (*N* = 392)	NSAI (*n* = 336)	SAI (*n* = 56)	Statistic	*p*
Age, Mean ± SD	60.63 ± 12.80	59.05 ± 12.00	70.14 ± 13.38	*t* = −6.30	**<0.001**
Height, Mean ± SD	167.36 ± 6.50	167.71 ± 6.40	165.27 ± 6.79	*t* = 2.62	**0.009**
Weight, Mean ± SD	69.16 ± 9.76	69.87 ± 9.60	64.90 ± 9.76	*t* = 3.58	**<0.001**
BMI, Mean ± SD	24.61 ± 2.87	24.77 ± 2.88	23.60 ± 2.57	*t* = 2.86	**0.004**
SBP, Mean ± SD	154.25 ± 22.29	153.65 ± 22.40	157.86 ± 21.50	*t* = −1.31	0.191
DBP, Mean ± SD	89.02 ± 15.39	89.73 ± 15.73	84.77 ± 12.40	*t* = 2.25	**0.025**
NIHSS_add, M (Q₁, Q₃)	3.00 (1.00, 4.25)	2.00 (1.00, 4.00)	5.00 (3.75, 10.00)	*Z* = −5.85	**<0.001**
CRP, M (Q₁, Q₃)	1.13 (0.50, 2.71)	1.12 (0.50, 2.56)	1.79 (0.50, 4.82)	*Z* = −1.79	0.073
WBC, M (Q₁, Q₃)	7.38 (6.00, 9.23)	7.38 (5.98, 9.31)	7.38 (6.24, 8.91)	*Z* = −0.13	0.894
RBC, M (Q₁, Q₃)	4.83 (4.48, 5.19)	4.84 (4.53, 5.20)	4.75 (4.25, 5.11)	*Z* = −2.08	**0.038**
HGB, M (Q₁, Q₃)	148.00 (137.00, 160.00)	148.00 (138.00, 160.00)	144.50 (127.00, 154.50)	*Z* = −1.93	0.054
PLT, M (Q₁, Q₃)	214.00 (179.75, 251.50)	216.00 (185.75, 256.00)	203.50 (174.00, 220.50)	*Z* = −2.49	**0.013**
WBC 2, M (Q₁, Q₃)	4.89 (3.73, 6.38)	4.77 (3.70, 6.38)	5.18 (3.93, 6.37)	*Z* = −0.64	0.519
N, M (Q₁, Q₃)	0.40 (0.32, 0.50)	0.40 (0.32, 0.50)	0.42 (0.34, 0.53)	*Z* = −1.14	0.254
L, M (Q₁, Q₃)	1.69 (1.33, 2.30)	1.71 (1.35, 2.30)	1.55 (1.18, 2.30)	*Z* = −1.65	0.100
PDW, M (Q₁, Q₃)	11.50 (10.50, 12.80)	11.50 (10.50, 12.80)	11.60 (10.40, 12.90)	*Z* = −0.14	0.889
SIRI, M (Q₁, Q₃)	1.14 (0.73, 1.77)	1.10 (0.69, 1.67)	1.26 (0.90, 2.24)	*Z* = −1.94	0.053
NLR, M (Q₁, Q₃)	2.74 (1.84, 4.14)	2.69 (1.81, 3.96)	3.31 (1.96, 4.98)	*Z* = −1.73	0.083
ALT, M (Q₁, Q₃)	22.00 (16.00, 29.00)	22.00 (16.00, 31.00)	20.00 (16.00, 24.25)	*Z* = −1.09	0.277
AST, M (Q₁, Q₃)	25.00 (22.00, 30.00)	25.00 (21.00, 30.00)	26.00 (23.75, 28.25)	*Z* = −0.82	0.410
ALP, M (Q₁, Q₃)	82.00 (70.00, 95.00)	82.00 (70.00, 94.00)	85.50 (73.00, 101.50)	*Z* = −1.07	0.285
LDH, M (Q₁, Q₃)	201.70 (176.82, 231.05)	202.00 (177.00, 230.02)	200.03 (173.50, 236.06)	*Z* = −0.01	0.992
TP, M (Q₁, Q₃)	72.52 (68.00, 77.00)	72.51 (68.00, 77.00)	72.70 (68.00, 76.00)	*Z* = −0.41	0.680
Alb, M (Q₁, Q₃)	43.97 (40.16, 46.00)	44.00 (40.69, 46.33)	41.95 (39.00, 44.16)	*Z* = −2.79	**0.005**
BIL, M (Q₁, Q₃)	13.35 (11.00, 16.15)	13.90 (11.00, 16.33)	11.97 (10.00, 15.85)	*Z* = −1.68	0.092
Cr, M (Q₁, Q₃)	73.00 (60.95, 86.00)	73.00 (61.00, 84.93)	76.50 (58.42, 94.25)	*Z* = −0.71	0.479
BUN, M (Q₁, Q₃)	5.80 (4.80, 6.80)	5.73 (4.80, 6.70)	6.15 (5.20, 7.75)	*Z* = −1.86	0.063
UA, M (Q₁, Q₃)	341.73 (291.75, 396.25)	342.00 (294.99, 394.25)	336.07 (250.75, 409.75)	*Z* = −0.89	0.371
NIHSS_five, M (Q₁, Q₃)	2.00 (1.00, 4.00)	2.00 (1.00, 3.02)	6.50 (3.00, 10.00)	*Z* = −7.96	**<0.001**
TC, M (Q₁, Q₃)	4.77 (4.20, 5.57)	4.76 (4.21, 5.55)	4.96 (3.98, 5.73)	*Z* = −0.16	0.870
TG, M (Q₁, Q₃)	1.37 (1.05, 1.80)	1.38 (1.07, 1.83)	1.29 (0.93, 1.63)	*Z* = −1.56	0.118
HDL, M (Q₁, Q₃)	1.08 (0.94, 1.25)	1.08 (0.94, 1.25)	1.15 (0.95, 1.27)	*Z* = −0.47	0.636
LDL, M (Q₁, Q₃)	3.06 (2.59, 3.70)	3.05 (2.61, 3.65)	3.17 (2.48, 3.83)	*Z* = −0.21	0.837
VLDL, M (Q₁, Q₃)	389.50 (290.31, 504.09)	389.26 (291.61, 500.56)	392.24 (272.75, 552.36)	*Z* = −0.02	0.986
Lipoproteins a, M (Q₁, Q₃)	16.20 (10.90, 27.02)	15.80 (10.57, 26.20)	21.30 (12.25, 36.42)	*Z* = −2.04	**0.041**
APOAAPOB, M (Q₁, Q₃)	1.19 (0.99, 1.45)	1.19 (1.00, 1.45)	1.17 (0.98, 1.44)	*Z* = −0.43	0.668
Homocysteine, M (Q₁, Q₃)	14.00 (11.20, 18.67)	13.80 (11.17, 18.28)	15.45 (11.80, 20.23)	*Z* = −1.55	0.120
BG_second, M (Q₁, Q₃)	6.00 (5.23, 7.57)	5.87 (5.19, 7.27)	7.32 (5.61, 9.22)	*Z* = −3.28	**0.001**
AFP, M (Q₁, Q₃)	2.78 (2.04, 3.66)	2.79 (2.05, 3.76)	2.75 (1.87, 3.30)	*Z* = −1.48	0.138
CEA, M (Q₁, Q₃)	2.61 (1.69, 3.58)	2.51 (1.68, 3.50)	3.10 (2.04, 3.89)	*Z* = −1.84	0.066
CA125, M (Q₁, Q₃)	9.46 (6.65, 12.93)	8.93 (6.63, 12.45)	11.36 (7.53, 14.83)	*Z* = −2.54	**0.011**
CA153, M (Q₁, Q₃)	9.88 (6.97, 13.76)	9.86 (6.98, 13.52)	10.87 (6.82, 15.86)	*Z* = −0.87	0.385
CA199, M (Q₁, Q₃)	8.70 (4.82, 14.88)	8.66 (4.62, 14.64)	8.94 (6.83, 16.24)	*Z* = −1.40	0.160
CA724, M (Q₁, Q₃)	1.49 (1.04, 2.78)	1.53 (1.03, 3.02)	1.33 (1.06, 2.03)	*Z* = −1.38	0.167
NSE, M (Q₁, Q₃)	15.29 (12.77, 18.43)	15.11 (12.68, 17.79)	17.59 (14.68, 20.94)	*Z* = −3.51	**<0.001**
CRFRA21 1, M (Q₁, Q₃)	2.74 (2.04, 3.62)	2.70 (2.02, 3.54)	2.96 (2.22, 3.81)	*Z* = −1.52	0.129
ProGRP, M (Q₁, Q₃)	39.81 (31.36, 50.97)	39.36 (30.96, 48.64)	46.20 (35.67, 59.07)	*Z* = −2.73	**0.006**
SCC, M (Q₁, Q₃)	1.04 (0.75, 1.40)	1.00 (0.74, 1.33)	1.25 (0.97, 1.86)	*Z* = −3.72	**<0.001**
FT3, M (Q₁, Q₃)	4.06 (3.70, 4.58)	4.16 (3.79, 4.62)	3.70 (3.04, 4.01)	*Z* = −5.23	**<0.001**
FT4, M (Q₁, Q₃)	16.14 (14.79, 17.39)	16.20 (14.90, 17.33)	15.68 (14.47, 18.20)	*Z* = −0.68	0.495
TSH, M (Q₁, Q₃)	1.75 (1.09, 2.73)	1.74 (1.08, 2.70)	1.76 (1.16, 3.03)	*Z* = −0.78	0.436
Folate, M (Q₁, Q₃)	8.30 (5.58, 11.45)	8.27 (5.42, 11.45)	8.57 (5.89, 11.54)	*Z* = −1.05	0.294
B12, M (Q₁, Q₃)	261.21 (174.54, 358.02)	262.15 (183.28, 367.40)	235.13 (147.25, 321.59)	*Z* = −1.60	0.110
AC, M (Q₁, Q₃)	−6.52 (−8.34, −5.10)	−6.93 (−8.52, −5.53)	−4.47 (−5.97, −3.23)	*Z* = −6.62	**<0.001**
DC, M (Q₁, Q₃)	6.15 (4.81, 7.73)	6.52 (5.22, 7.91)	4.25 (3.00, 5.57)	*Z* = −6.89	**<0.001**
SDNN, M (Q₁, Q₃)	100.00 (82.00, 123.00)	103.00 (85.00, 125.00)	78.50 (67.75, 101.00)	*Z* = −4.93	**<0.001**
SDANN, M (Q₁, Q₃)	80.00 (64.00, 99.00)	83.00 (67.75, 101.00)	63.00 (48.00, 78.25)	*Z* = −5.76	**<0.001**
RMSSD, M (Q₁, Q₃)	29.00 (21.00, 46.00)	29.00 (22.00, 44.00)	28.50 (18.00, 51.50)	*Z* = −0.08	0.937
PNN50, M (Q₁, Q₃)	4.00 (1.00, 9.00)	4.00 (1.00, 10.00)	2.00 (0.00, 6.75)	*Z* = −1.96	0.050
VLF, M (Q₁, Q₃)	1576.15 (926.42, 2490.00)	1669.20 (1076.30, 2568.15)	716.00 (372.07, 1471.03)	*Z* = −5.78	**<0.001**
LF, M (Q₁, Q₃)	318.25 (179.57, 562.40)	351.05 (202.17, 584.62)	169.30 (78.12, 372.75)	*Z* = −4.72	**<0.001**
HF, M (Q₁, Q₃)	112.75 (58.20, 209.62)	118.95 (69.67, 214.35)	48.65 (25.50, 138.92)	*Z* = −3.97	**<0.001**
LH/HF, M (Q₁, Q₃)	2.79 (1.78, 4.16)	2.81 (1.84, 4.10)	2.48 (1.51, 4.23)	*Z* = −1.28	0.201
DR, M (Q₁, Q₃)	−0.26 (−0.59, −0.06)	−0.29 (−0.60, −0.09)	−0.06 (−0.40, 0.07)	*Z* = −3.89	**<0.001**
Gender, *n* (%)				χ^2^ = 3.38	0.066
Male	291 (74.23)	255 (75.89)	36 (64.29)		
Female	101 (25.77)	81 (24.11)	20 (35.71)		
Diabetes, *n* (%)				χ^2^ = 2.83	0.093
No	288 (73.47)	252 (75.00)	36 (64.29)		
Yes	104 (26.53)	84 (25.00)	20 (35.71)		
Hypertension, *n* (%)				χ^2^ = 6.97	**0.008**
No	161 (41.07)	147 (43.75)	14 (25.00)		
Female	231 (58.93)	189 (56.25)	42 (75.00)		
CHD, *n* (%)				χ^2^ = 0.27	0.604
No	379 (96.68)	326 (97.02)	53 (94.64)		
Yes	13 (3.32)	10 (2.98)	3 (5.36)		
Stroke, *n* (%)				χ^2^ = 4.56	**0.033**
No	353 (90.05)	307 (91.37)	46 (82.14)		
Yes	39 (9.95)	29 (8.63)	10 (17.86)		
Smoke, *n* (%)				χ^2^ = 1.74	0.188
No	318 (81.12)	269 (80.06)	49 (87.50)		
Yes	74 (18.88)	67 (19.94)	7 (12.50)		
Alcohol, *n* (%)				χ^2^ = 2.13	0.145
No	340 (86.73)	288 (85.71)	52 (92.86)		
Yes	52 (13.27)	48 (14.29)	4 (7.14)		
Thrombolysis, *n* (%)				χ^2^ = 1.71	0.192
No	337 (85.97)	292 (86.90)	45 (80.36)		
Yes	55 (14.03)	44 (13.10)	11 (19.64)		
Bleeding, *n* (%)				χ^2^ = 21.55	**<0.001**
No	382 (97.45)	333 (99.11)	49 (87.50)		
Yes	10 (2.55)	3 (0.89)	7 (12.50)		
NG, *n* (%)				χ^2^ = 123.20	**<0.001**
No	358 (91.33)	329 (97.92)	29 (51.79)		
Yes	34 (8.67)	7 (2.08)	27 (48.21)		
UC, *n* (%)				χ^2^ = 104.05	**<0.001**
No	361 (92.09)	329 (97.92)	32 (57.14)		
Yes	31 (7.91)	7 (2.08)	24 (42.86)		
TOAST, *n* (%)				−	**<0.001**
LAA	122 (31.12)	90 (26.79)	32 (57.14)		
CE	204 (52.04)	182 (54.17)	22 (39.29)		
SAA	16 (4.08)	15 (4.46)	1 (1.79)		
SOE	5 (1.28)	5 (1.49)	0 (0.00)		
SUE	45 (11.48)	44 (13.10)	1 (1.79)		

*t*, *t*-test; *Z*, Mann–Whitney test; χ^2^, chi-squared test; −, Fisher exact.

SD, standard deviation; M, Median; Q₁, 1st Quartile; Q₃, 3rd Quartile.

The full names and abbreviations of the included features are listed in Supplementary Table S1.

The bold values denote less than 0.05.

### 3.2 Variable selection

**Figure 2:**
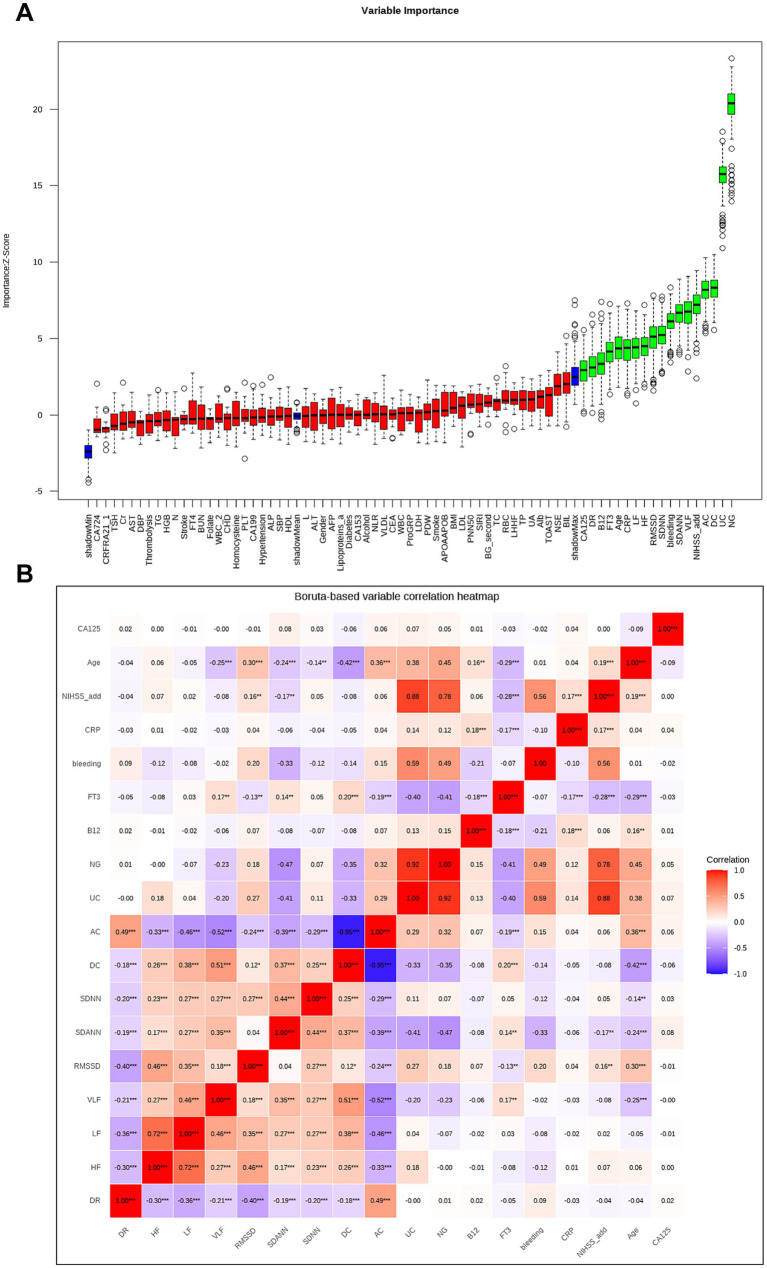
Predictor screening results: **(A)** Boruta; **(B)** Boruta-based variable correlation heatmap. *** Indicates that the significance level of the correlation coefficient is less than 0.001; ** indicates that the significance level of the correlation coefficient is less than 0.01; * indicates that the significance level of the correlation coefficient is less than 0.05.

To determine the variables for inclusion in the machine learning models, the Boruta algorithm assessed differences in various indicators between patients with and without SAI. The Boruta algorithm identified 18 key factors, including NG, UC, DC, AC, NIHSS_add, VLF, SDANN, bleeding, SDNN, RMSSD, HF, LF, CRP, Age, FT3, B12, DR, and CA125 (Figure 2A). Spearman correlation analysis was performed on the selected variables, revealing three pairs of highly correlated variables: AC with DC (*ρ* = −0.946), NG with UC (*ρ* = 0.921), and NIHSS_add with UC (*ρ* = 0.886) (Figure 2B). Based on variable importance scores and clinical significance, we retained DC (due to its superiority in predicting heart rate variability prognosis) (21), NG (for its specificity in brainstem function), and NIHSS score (the gold standard indicator of stroke severity), while excluding AC and UC. The final optimized feature set comprising 16 relatively independent variables was obtained for subsequent modeling.

### 3.3 Development and evaluation of the SAI diagnostic model

**Figure 3:**
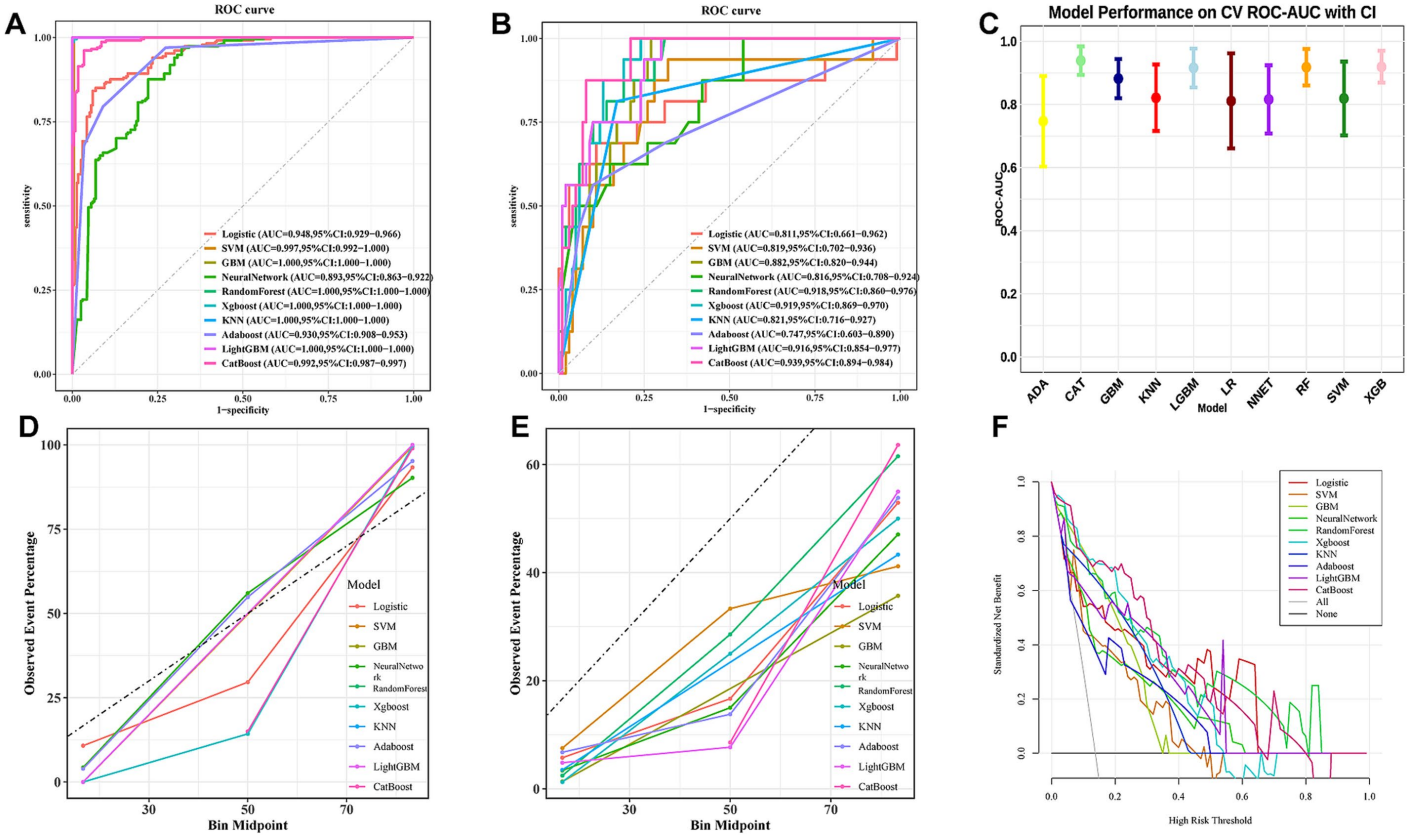
The performance and comparison of 10 different predictive models. **(A)** The training set ROC curve; **(B)** the test set ROC curve: **(C)** model performance on 10-fold CV ROC-AUC with CI; **(D)** calibration curve of the training set; **(E)** calibration curve of the test set; **(F)** decision curve analysis of 10 different predictive models for the test set. LR, Logistic Regression; SVM, Support Vector Machine; GBM, Gradient Boosting Machine; XGB, Extreme Gradient Boosting; KNN, *K*-Nearest Neighbors; ADA, Adaptive Boosting; LGBM, Light Gradient Boosting Machine; CAT, Categorical Boosting; CI, Confidence Interval.

In the model training, a positive class represented the presence of SAI, while a negative class represented the absence of SAI. Utilizing 16 features, we developed 10 different machine learning models, including GBM, RF, LR, SVM, KNN, NNET, CAT, ADA, LGBM, and XGB. In the training set, the GBM, RF, XGB, KNN, and LGBM models exhibited superior predictive performance with an AUC of 1.00, indicating a high level of accuracy in prediction. In contrast, the AUC values for the remaining three models were as follows: 0.948, 95% CI (0.929–0.966) for LR, 0.997, 95% CI (0.992–1.000) for SVM, 0.893, 95% CI (0.863–0.922) for NNET, 0.930, 95% CI (0.908–0.953) for ADA, and 0.992,95% CI (0.987–0.997) for CAT (Figure 3A). In the test set, the findings of this study demonstrate that the CAT model displayed a significantly higher AUC value in comparison to other machine learning algorithms (Figure 3B). The model performance plot comparing the AUC scores of the 10 machine learning models is presented (Figure 3C). In this study, the accuracy, sensitivity, specificity, positive predictive value, negative predictive value and F1 score of each model were computed and compared (Table 2). Further examination of the data in the internal validation cohort revealed that the CAT model exhibited an accuracy of 0.914, a sensitivity of 0.875, a specificity of 0.92, an F1 score of 0.737, and an AUC value of 0.939 (Table 2). The calibration curve results for each model on the training and test sets are shown in Figures 3D,E. While the GBM, RF, XGB, KNN, and LGBM models exhibited exceptional performance on the training set, the CAT model was ultimately selected as the optimal model due to concerns regarding potential overfitting and the fact that the CAT model had the highest AUC value on the test set. DCA is a straightforward method to evaluate the clinical utility of disease diagnostic models. The DCA curve depicted in Figure 3F further demonstrated that the CAT model had higher clinical utility than other models.

**Table 2:** Diagnostic performance of each model in test set.

Model	Accuracy	Sensitivity	Specificity	Precision	F1	AUC
LR	0.862	0.688	0.89	0.500	0.579	0.811
SVM	0.716	0.938	0.68	0.319	0.476	0.819
GBM	0.767	1.000	0.73	0.372	0.542	0.882
NNET	0.819	0.625	0.85	0.400	0.488	0.816
RF	0.733	1.000	0.69	0.34	0.508	0.918
XGB	0.793	1.000	0.76	0.400	0.571	0.919
KNN	0.828	0.812	0.83	0.433	0.565	0.821
ADA	0.853	0.562	0.90	0.474	0.514	0.747
LGBM	0.741	1.000	0.70	0.348	0.516	0.916
CAT	0.914	0.875	0.92	0.636	0.737	0.939

LR, Logistic Regression; SVM, Support Vector Machine; GBM, Gradient Boosting Machine; XGB, Extreme Gradient Boosting; KNN, K-Nearest Neighbors; ADA, Adaptive Boosting; LGBM, Light Gradient Boosting Machine; CAT, Categorical Boosting.

### 3.4 Model interpretation based on SHAP

**Figure 4:**
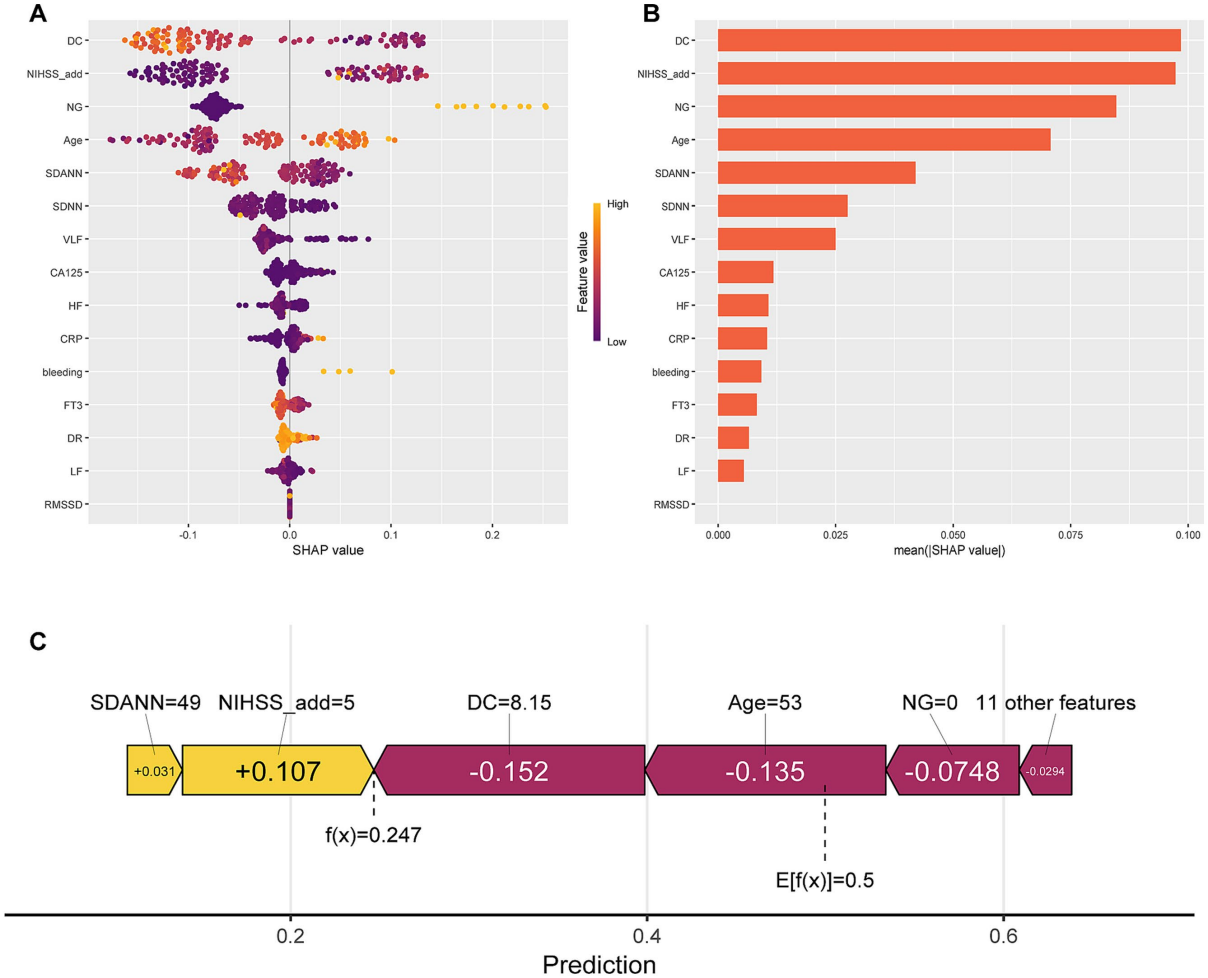
Interpretability analysis of the categorical boosting (CAT) model. **(A)** SHAP dendrogram of features of the CAT model. **(B)** Importance ranking plot of features of the CAT model. **(C)** Personalized predictions for a patient. Higher functional significance is indicated by longer bars. The full names and abbreviations of the included features are listed in Supplementary Table S1.

SHAP are popular model interpretability frameworks featuring various approaches. In addition, SHAP offers global and local insights with dual interpretability (22). To elucidate the predictive significance of selected variables within the optimal CAT model for SAI, we implemented the SHAP for comprehensive feature interpretation. Figure 4A presents a visual representation of the 15 pivotal features in the CAT model, where individual data points are color-coded to reflect their risk associations: yellow hues denote elevated risk values, while purple shades indicate reduced risk values. Figure 4B illustrates the hierarchical clustering of these 15 risk factors, emphasizing their relative contributions to the model’s predictive capability. The SHAP values, plotted along the x-axis, quantitatively demonstrate the magnitude of each factor’s influence. The robust correlation between these 15 indicators and the underlying SAI pathogenesis underscores their potential utility as reliable biomarkers for clinical detection and monitoring of disease progression. Shapley values identified the most informative features as DC, NIHSS_add, NG, and Age, while SDANN, SDNN, VLF, CA125, HF, CRP, bleeding, FT3, DR, LF, and RMSSD only had a marginal impact on model classification (Figure 4B). Meanwhile, Figure 4C displays the SHAP explanation force diagram from the CAT model’s test set. Red bars showed that the listed characteristics decreased the occurrence of SAI, whereas the yellow bars indicate the opposite.

## 4 Discussion

SAI is a significant risk factor for poor prognosis in patients with AIS. Early clinical diagnosis and intervention are crucial for reducing the incidence of SAI and improving patients’ quality of life. To identify high-risk populations, this study incorporated PRSA parameters related to autonomic nervous function into the model. We established the first CAT model based on PRSA indicators for the early diagnosis and progression monitoring of SAI. The CAT model, composed of clinical data and PRSA parameters, enables non-invasive and reliable diagnosis of SAI. Furthermore, the CAT model can predict the occurrence of SAI in advance. The findings of this study hold potential significance for predicting SAI early, facilitating timely interventions, reducing its incidence, and ultimately improving patient outcomes.

The impact of changes in the autonomic nervous system after AIS on the immune system is well-documented. The vagus nerve can reduce the release of pro-inflammatory cytokines, such as TNF-*α*, through acetylcholine acting on α7nAChR, a signaling pathway considered critical for preventing excessive inflammation (23). In this study, we observed significant impairment in overall autonomic nervous system regulation in SAI patients compared to NSAI patients. Previous studies have also reported reduced parasympathetic regulation rather than activation in AIS patients compared to controls (14), with the vagus nerve exerting a protective effect in ischemic stroke (24, 25). This may be attributed to the parasympathetic innervation of the Circle of Willis and leptomeningeal arteries (26). Thus, parasympathetic activation induces arterial dilation, increasing blood flow to the affected regions. Additionally, the balance between the sympathetic and parasympathetic systems is essential for maintaining normal physiological functions. Impaired parasympathetic function may lead to relative sympathetic activation, which, post-AIS, can cause atrophy of immune organs and functional changes in immune cells, thereby suppressing systemic immune responses (27, 28).

This study employed a dual approach using the Boruta algorithm and correlation analysis to identify predictors, ensuring accurate feature selection and model stability. The Boruta algorithm, applied without pre-selection bias toward either PRSA or conventional variables, identified specific PRSA parameters as consistently significant predictors. This indicates that these parameters provide unique prognostic information that is not fully captured by standard clinical assessments alone. By integrating these novel parameters, our final model offers a more holistic risk assessment tool. Selected features included NG, DC, NIHSS_add, VLF, SDANN, bleeding, SDNN, RMSSD, HF, LF, CRP, Age, FT3, B12, DR, and CA125. Ten widely used machine learning algorithms were applied to analyze medical data and construct a predictive model for SAI. Given the retrospective nature of the study, we reported the missing rates for each variable (Supplementary Figure S1) and excluded variables with missing rates exceeding 20%. For variables with missing rates below 20%, imputation was performed using the missRanger package to enhance model robustness. Additionally, an advanced machine learning technique, such as SMOTE, was employed to address class imbalance. Among the evaluated models, including GBM, RF, XGB, KNN, and LGBM, several demonstrated superior performance on the training set. However, their perfect or near-perfect training scores (AUC = 1.00) raised concerns regarding potential overfitting. Consequently, the CAT model was selected as the optimal choice for the final model, as it demonstrated consistently strong and more reliable performance on the independent test set (AUC = 0.939), indicating superior generalizability for clinical use. DCA curves in Figure 3F further confirmed the higher clinical utility of the CAT model.

The importance of constructing disease prediction models lies in identifying high-risk patients and mitigating risks for individuals likely to belong to this group, thereby benefiting the overall patient population. Consequently, the clinical interpretability of machine learning models is of paramount value in medical practice. To address this, SHAP was utilized to enhance model transparency and interpretability. As shown in Figure 4, this study identified factors closely associated with SAI, including Age, NG, DC, and NIHSS_add. Age, NIHSS_add, and NG have been reported to be among the independent risk factors for stroke-associated pneumonia in previous studies (29–31), but little research has been done on the relationship between DC or AC and SAI. In patients with AIS, the assessment of autonomic function is crucial for immune function. DC and AC are quantitative indices that capture the capacity for heart rate deceleration and acceleration. In our study, the SAI group exhibited significantly lower DC and less negative AC values compared to the NSAI group (Table 1). While this pattern is consistent with a shift in autonomic balance, it is crucial to interpret these findings within the methodological context of PRSA. As highlighted by Rivolta et al., the prognostic power of DC and AC may stem more from their sensitivity to non-stationarities and overall autonomic dysfunction than from their ability to discretely quantify vagal or sympathetic tone (13). The significant difference in DR between groups supports this interpretation, as DR is designed to be insensitive to the overall signal power and specifically captures the asymmetry between deceleration and acceleration capacities, which is a hallmark of pathological autonomic regulation (13) (Table 1). However, DR did not perform well in the SHAP analysis of this study. Therefore, the combined use of DC, AC, and particularly DR provides a more comprehensive assessment of the altered autonomic state post-stroke, reflecting overall regulatory impairment rather than isolated branch function.

The greatest strength of this paper is the first use of parameters related to PRSA technology to construct a predictive model for post-stroke infection. Quantifying autonomic nervous system function is challenging. In previous studies, HRV parameters, such as SDNN, LF, and RMSDD, have often been used to quantify autonomic nervous system function. However, HRV is affected by both vagal and sympathetic modulation of the sinus node, and it is not possible to differentiate between vagal and sympathetic roles (15). In addition, HRV is affected by a variety of factors (32). The PRSA technique has been suggested to be a better method to quantify autonomic function, allowing the analysis of vagal and sympathetic activity by DC and AC. Its specificity and sensitivity have been shown to predict mortality after myocardial infarction is confirmed (15). DC and AC are innovative indicators of the autonomic nervous system. They utilize signal processing algorithms to distinguish between deceleration and acceleration of heart rate as a metric of cardiac autonomic regulation. DC and AC techniques have advantages over traditional techniques such as HRV. First, they are able to quantitatively assess a patient’s autonomic activity. Second, AC and DC, calculated by PRSA, are less susceptible to noise interference and have better sensitivity, specificity, and stability than HRV (33). Together, AC and DC constitute a “bi-directional indicator” of heart rate regulation. The dynamic balance between the two maintains cardiovascular homeostasis, and an abnormal AC/DC ratio may reflect dysregulation of the sympathovagal balance. For example, a decrease in AC accompanied by an increase in DC suggests a predominance of vagal tone, which is commonly seen in patients with vasovagal syncope (33).

Given that SAP is the most prevalent form of SAI (34), prior research has focused on identifying biomarkers for SAP prediction (35, 36), such as immune, inflammatory, and stress-related proteins, as well as ratios and indices such as the neutrophil-to-lymphocyte ratio (NLR), systemic immune-inflammation index (SII), platelet-to-lymphocyte ratio (PLR), and systemic inflammation response index (SIRI). Among these, NLR has been reported as the best predictor of SAP (37). HRV, particularly very low-frequency HRV (38, 39), a composite indicator of autonomic and humoral control, has been identified as an early marker for post-stroke infections. However, these biomarkers either failed to pass the variable selection in this study or provided only marginal improvements in the prediction of post-stroke infections (39, 40). Nelde et al. developed an LR model to predict stroke-associated pneumonia in stroke patients (34), incorporating HRV parameters based on prior research. Their results indicated that most HRV parameters were poor predictors of SAP, consistent with our findings. However, conventional clinical parameters (e.g., CRP and WBC) showed significant importance in their SAP prediction model, diverging from our results. This discrepancy may stem from differences in variable selection and study outcomes, highlighting the need for novel and more reliable predictive indicators.

This study has several limitations. First, due to the limited dataset from the electronic medical records of Shanghai Sixth People’s Hospital, we were unable to separately analyze PRSA in relation to SAP, UTI, and other infections occurring within a week post-stroke. Also, while our study utilized the default PRSA parameters (T = 1, s = 2, L = 50), we acknowledge that these values may not be optimal for AIS populations. PRSA parameters are highly application-specific; for instance, studies in fetal heart rate analysis (13) and other conditions have shown that tuning T, s, and L can enhance sensitivity to specific autonomic patterns. Our choice of parameters was based on consistency with prior cardiovascular research (15), ensuring comparability, but it may not fully capture the unique autonomic dysfunction in AIS. Therefore, parameter optimization represents a critical direction for future research. Also, we acknowledge that the exclusion of patients with atrial fibrillation may affect the immediate generalizability of our model to all stroke populations; future studies are warranted to validate and potentially adapt the model for cohorts with significant arrhythmias. Finally, the future clinical applicability of the model requires external prospective validation.

## 5 Conclusion

We developed and validated an interpretable machine learning model to assess risk factors for SAI in patients with AIS. First, the model can rapidly identify patients at higher risk of infection based on available variables. Additionally, we identified Age, NG, DC, and NHISS_add as significant risk factors in the study population. Finally, SHAP was employed to interpret the predictive model, enhancing its interpretability and clinical utility. SAI is associated with increased mortality, prolonged hospitalization, and the need for long-term rehabilitation and care, thereby imposing greater familial and healthcare burdens. In this context, we propose that the PRSA markers analyzed here could serve as potential targets for preventive interventions, enabling more judicious, timely, and targeted use of antibiotics. This approach opens new avenues for research into the prophylactic management of SAI.

## Data Availability

The raw data supporting the conclusions of this article will be made available by the authors, without undue reservation.

## References

[1] Feske SK. Ischemic stroke. Am J Med. (2021) 134:1457–64. doi: 10.1016/j.amjmed.2021.07.027, 34454905

[2] Adams HP Bendixen BH Kappelle LJ Biller J Love BB Gordon DL . Classification of subtype of acute ischemic stroke. Definitions for use in a multicenter clinical trial. TOAST. trial of org 10172 in acute stroke treatment. Stroke. (1993) 24:35–41. doi: 10.1161/01.STR.24.1.35, 7678184

[3] Barthels D Das H. Current advances in ischemic stroke research and therapies. Biochim Biophys Acta Mol basis Dis. (2020) 1866:165260. doi: 10.1016/j.bbadis.2018.09.012, 31699365 PMC6981280

[4] Suda S Aoki J Shimoyama T Suzuki K Sakamoto Y Katano T . Stroke-associated infection independently predicts 3-month poor functional outcome and mortality. J Neurol. (2018) 265:370–5. doi: 10.1007/s00415-017-8714-6, 29249057

[5] Chen X Liang X Zhang J Chen L Sun J Cai X. Serum calcium levels and in-hospital infection risk in patients with acute ischemic stroke. Neuropsychiatr Dis Treat. (2022) 18:943–50. doi: 10.2147/NDT.S354447, 35535212 PMC9078440

[6] Spratt N Wang Y Levi C Ng K Evans M Fisher J. A prospective study of predictors of prolonged hospital stay and disability after stroke. J Clin Neurosci. (2003) 10:665–9. doi: 10.1016/j.jocn.2002.12.001, 14592613

[7] Katzan IL Cebul RD Husak SH Dawson NV Baker DW. The effect of pneumonia on mortality among patients hospitalized for acute stroke. Neurology. (2003) 60:620–5. doi: 10.1212/01.WNL.0000046586.38284.60, 12601102

[8] Wästfelt M Cao Y Ström JO. Predictors of post-stroke fever and infections: a systematic review and meta-analysis. BMC Neurol. (2018) 18:49. doi: 10.1186/s12883-018-1046-z, 29685118 PMC5913801

[9] Xiong L Tian G Leung H Soo YOY Chen X Ip VHL . Autonomic dysfunction predicts clinical outcomes after acute ischemic stroke: a prospective observational study. Stroke. (2018) 49:215–8. doi: 10.1161/STROKEAHA.117.019312, 29203690

[10] Wang Y-Y Lin S-Y Chuang Y-H Sheu WH-H Tung K-C Chen C-J. Activation of hepatic inflammatory pathways by catecholamines is associated with hepatic insulin resistance in male ischemic stroke rats. Endocrinology. (2014) 155:1235–46. doi: 10.1210/en.2013-1593, 24437486

[11] Yuan M Han B Xia Y Liu Y Wang C Zhang C. Augmentation of peripheral lymphocyte-derived cholinergic activity in patients with acute ischemic stroke. BMC Neurol. (2019) 19:236. doi: 10.1186/s12883-019-1481-5, 31615442 PMC6792255

[12] Wang X-D Zhou L Zhu C-Y Chen B Chen Z Wei L. Autonomic function as indicated by heart rate deceleration capacity and deceleration runs in type 2 diabetes patients with or without essential hypertension. Clin Interv Aging. (2018) 13:1169–76. doi: 10.2147/CIA.S149920, 29997434 PMC6033089

[13] Rivolta MW Stampalija T Frasch MG Sassi R. Theoretical value of deceleration capacity points to deceleration reserve of fetal heart rate. IEEE Trans Biomed Eng. (2020) 67:1176–85. doi: 10.1109/TBME.2019.2932808, 31395532

[14] Xu Y-H Wang X-D Yang J-J Zhou L Pan Y-C. Changes of deceleration and acceleration capacity of heart rate in patients with acute hemispheric ischemic stroke. Clin Interv Aging. (2016) 11:293–8. doi: 10.2147/CIA.S99542, 27042028 PMC4795583

[15] Bauer A Kantelhardt JW Barthel P Schneider R Mäkikallio T Ulm K . Deceleration capacity of heart rate as a predictor of mortality after myocardial infarction: cohort study. Lancet. (2006) 367:1674–81. doi: 10.1016/S0140-6736(06)68735-7, 16714188

[16] Benichou T Pereira B Mermillod M Tauveron I Pfabigan D Maqdasy S . Heart rate variability in type 2 diabetes mellitus: a systematic review and meta-analysis. PLoS One. (2018) 13:e0195166. doi: 10.1371/journal.pone.0195166, 29608603 PMC5880391

[17] Otzenberger H Gronfier C Simon C Charloux A Ehrhart J Piquard F . Dynamic heart rate variability: a tool for exploring sympathovagal balance continuously during sleep in men. Am J Phys. (1998) 275:H946–50. doi: 10.1152/ajpheart.1998.275.3.H946, 9724299

[18] Wright MN Ziegler A. Ranger: a fast implementation of random forests for high dimensional data in C++ and R. J Stat Softw. (2017) 77:1–17. doi: 10.18637/jss.v077.i01

[19] Roberts GW Quinn SJ Valentine N Alhawassi T O’Dea H Stranks SN . Relative hyperglycemia, a marker of critical illness: introducing the stress hyperglycemia ratio. J Clin Endocrinol Metabol. (2015) 100:4490–7. doi: 10.1210/jc.2015-2660, 26485219

[20] Yu M Yuan Z Li R Shi B Wan D Dong X. Interpretable machine learning model to predict surgical difficulty in laparoscopic resection for rectal cancer. Front Oncol. (2024) 14:1337219. doi: 10.3389/fonc.2024.1337219, 38380369 PMC10878416

[21] Zhou H Zhong J Deng C Wang X Xu Y Yang J. Prognostic value of heart rate deceleration capacity for functional outcomes in acute ischemic stroke: a prospective study. Front Endocrinol. (2025) 16:1601346. doi: 10.3389/fendo.2025.1601346, 40453588 PMC12123432

[22] Vimbi V Shaffi N Mahmud M. Interpreting artificial intelligence models: a systematic review on the application of LIME and SHAP in alzheimer’s disease detection. Brain Inform. (2024) 11:10. doi: 10.1186/s40708-024-00222-1, 38578524 PMC10997568

[23] Tracey KJ. Physiology and immunology of the cholinergic antiinflammatory pathway. J Clin Invest. (2007) 117:289–96. doi: 10.1172/JCI30555, 17273548 PMC1783813

[24] Wang Y-Y Lin S-Y Chang C-Y Wu C-C Chen W-Y Huang W-C . α7 nicotinic acetylcholine receptor agonist improved brain injury and impaired glucose metabolism in a rat model of ischemic stroke. Metab Brain Dis. (2023) 38:1249–59. doi: 10.1007/s11011-023-01167-w, 36662413

[25] Suzuki N Hardebo JE Kåhrström J Owman C. Selective electrical stimulation of postganglionic cerebrovascular parasympathetic nerve fibers originating from the sphenopalatine ganglion enhances cortical blood flow in the rat. J Cereb Blood Flow Metab. (1990) 10:383–91. doi: 10.1038/jcbfm.1990.68, 2329125

[26] Suzuki N Hardebo JE Owman C. Origins and pathways of cerebrovascular vasoactive intestinal polypeptide-positive nerves in rat. J Cereb Blood Flow Metab. (1988) 8:697–712. doi: 10.1038/jcbfm.1988.117, 3417797

[27] Offner H Subramanian S Parker SM Wang C Afentoulis ME Lewis A . Splenic atrophy in experimental stroke is accompanied by increased regulatory T cells and circulating macrophages. J Immunol. (2006) 176:6523–31. doi: 10.4049/jimmunol.176.11.6523, 16709809

[28] Liu Q Jin WN Liu Y Shi K Sun H Zhang F . Brain ischemia suppresses immunity in the periphery and brain via different neurogenic innervations. Immunity. (2017) 46:474–87. doi: 10.1016/j.immuni.2017.02.015 28314594

[29] Liu F. Analysis of risk factors for pulmonary infection in acute ischemic stroke patients following intravenous thrombolysis with alteplase. Am J Transl Res. (2024) 16:4643–52. doi: 10.62347/VZQQ5140, 39398567 PMC11470298

[30] Wen SW Shim R Ho L Wanrooy BJ Srikhanta YN Prame Kumar K . Advanced age promotes colonic dysfunction and gut-derived lung infection after stroke. Aging Cell. (2019) 18:e12980. doi: 10.1111/acel.12980, 31199577 PMC6718525

[31] Yuan M Li F Tian X Wang W Jia M Wang X . Risk factors for lung infection in stroke patients: a meta-analysis of observational studies. Expert Rev Anti-Infect Ther. (2015) 13:1289–98. doi: 10.1586/14787210.2015.1085302, 26359533

[32] Onishi Y Minoura Y Chiba Y Onuki T Ito H Adachi T . Daily dysfunction of autonomic regulation based on ambulatory blood pressure monitoring in patients with neurally mediated reflex syncope. Pacing Clin Electrophysiol. (2015) 38:997–1004. doi: 10.1111/pace.12661, 25974151

[33] Zheng L Sun W Liu S Liang E Du Z Guo J . The diagnostic value of cardiac deceleration capacity in vasovagal syncope. Circ Arrhythm Electrophysiol. (2020) 13:e008659. doi: 10.1161/CIRCEP.120.008659, 33197331

[34] Nelde A Krumm L Arafat S Hotter B Nolte CH Scheitz JF . Machine learning using multimodal and autonomic nervous system parameters predicts clinically apparent stroke-associated pneumonia in a development and testing study. J Neurol. (2024) 271:899–908. doi: 10.1007/s00415-023-12031-3, 37851190 PMC10827826

[35] Westendorp WF Dames C Nederkoorn PJ Meisel A. Immunodepression, infections, and functional outcome in ischemic stroke. Stroke. (2022) 53:1438–48. doi: 10.1161/STROKEAHA.122.038867, 35341322

[36] Faura J Bustamante A Miró-Mur F Montaner J. Stroke-induced immunosuppression: implications for the prevention and prediction of post-stroke infections. J Neuroinflammation. (2021) 18:127. doi: 10.1186/s12974-021-02177-0, 34092245 PMC8183083

[37] Wang R-H Wen W-X Jiang Z-P Du Z-P Ma Z-H Lu A-L . The clinical value of neutrophil-to-lymphocyte ratio (NLR), systemic immune-inflammation index (SII), platelet-to-lymphocyte ratio (PLR) and systemic inflammation response index (SIRI) for predicting the occurrence and severity of pneumonia in patients with intracerebral hemorrhage. Front Immunol. (2023) 14:1115031. doi: 10.3389/fimmu.2023.1115031, 36860868 PMC9969881

[38] Günther A Salzmann I Nowack S Schwab M Surber R Hoyer H . Heart rate variability - a potential early marker of sub-acute post-stroke infections. Acta Neurol Scand. (2012) 126:189–96. doi: 10.1111/j.1600-0404.2011.01626.x, 22118023

[39] Brämer D Günther A Rupprecht S Nowack S Adam J Meyer F . Very low frequency heart rate variability predicts the development of post-stroke infections. Transl Stroke Res. (2019) 10:607–19. doi: 10.1007/s12975-018-0684-1, 30617993

[40] Hotter B Hoffmann S Ulm L Montaner J Bustamante A Meisel C . Inflammatory and stress markers predicting pneumonia, outcome, and etiology in patients with stroke: biomarkers for predicting pneumonia, functional outcome, and death after stroke. Neurol Neuroimmunol Neuroinflamm. (2020) 7:e692. doi: 10.1212/NXI.0000000000000692, 32098866 PMC7051196

