# Supplementary material for: Integrating phase-rectified signal averaging with machine learning to predict stroke-associated infections: a retrospective cohort study
Source: Front Neurol. 2026 Jan 13;16:1653947. doi: 10.3389/fneur.2025.1653947 (PMC12834720; doi:10.3389/fneur.2025.1653947)
Supplement: Supplementary file 1 [file Table_1.docx]

**Supplementary Table S1** The full names, types and abbreviations of the included features

| **SN** | **Predictor** | **Description** | **Types** | **Values** |
| --- | --- | --- | --- | --- |
| 1 | Gender | Sex of the patient | Categorical | 0 male  1 female |
| 2 | Hypertension | History of hypertension | Categorical | 0 No  1 Yes |
| 3 | NG | Nasogastric tube | Categorical | 0 No  1 Yes |
| 4 | CHD | History of Coronary Heart Disease | Categorical | 0 No  1 Yes |
| 5 | bleeding | Hemorrhagic transformation | Categorical | 0 No  1 Yes |
| 6 | TOAST | Trial of Org 10172 in Acute Stroke Treatment | Categorical | 1 LAA^*^  2 CE^*^  3 SAA^*^  4 SOE^*^  5 SUE^*^ |
| 7 | Stroke | History of stroke | Categorical | 0 No  1 Yes |
| 8 | UC | Urinary catheterization | Categorical | 0 No  1 Yes |
| 9 | Smoke | History of smoking | Categorical | 0 No  1 Yes |
| 10 | Thrombolysis | Thrombolysis treatment | Categorical | 0 No  1 Yes |
| 11 | Alcohol | History of alcohol consumption | Categorical | 0 No  1 Yes |
| 12 | Diabetes | History of diabetes | Categorical | 0 No  1 Yes |
| 13 | Age | Age of the patient (years) | Continuous | 30-92 |
| 14 | Height | Height of the patient (cm) | Continuous | 146-185 |
| 15 | Weight | Weight of the patient (kg) | Continuous | 45-105 |
| 16 | BMI | Body mass index (kg/m2) | Continuous | 16.71-35.49 |
| 17 | SBP | Systolic blood pressure | Continuous | 93-234 |
| 18 | DBP | diastolic blood pressure | Continuous | 48-163 |
| 19 | NIHSS_add | the National Institute of Health Stroke Scale at admission | Continuous | 0-28 |
| 20 | CRP | C—Reactive Protein | Continuous | 0.05-102.94 |
| 21 | WBC | White blood cell | Continuous | 3.44-230.09 |
| 22 | RBC | Red blood cell | Continuous | 2.88-15.20 |
| 23 | HGB | Hemoglobin | Continuous | 3-1170 |
| 24 | PLT | Platelet | Continuous | 81-2260 |
| 25 | N | Neutrophil | Continuous | 1.57-16.06 |
| **SN** | **Predictor** | **Description** | **Types** | **Values** |
| 26 | M | Monocyte | Continuous | 0.10-1.47 |
| 27 | L | Lymphocyte | Continuous | 0.29-7.29 |
| 28 | PDW | Platelet Distribution Width | Continuous | 0.00-22.00 |
| 29 | SIRI | Systemic Inflammatory Response Index | Continuous | 0.18-16.73 |
| 30 | NLR | Neutrophil-Lymphocyte count ratio | Continuous | 0.56-52.57 |
| 31 | ALT | Glutamic pyruvic  transaminase | Continuous | 6-118 |
| 32 | AST | Aspartate aminotransferase | Continuous | 11-96 |
| 33 | ALP | Alkaline phosphatase | Continuous | 11-423 |
| 34 | LDH | Lactate dehydrogenase | Continuous | 57-727 |
| 35 | TP | Total protein | Continuous | 39-101 |
| 36 | Alb | Albumin | Continuous | 18-74 |
| 37 | BIL | Bilirubin | Continuous | 3.0-18.4 |
| 38 | BG | Blood glucose | Continuous | 3.0-32.3 |
| 39 | Cr | Creatinine | Continuous | 38-269 |
| 40 | BUN | Blood urea nitrogen | Continuous | 2.7-16.5 |
| 41 | UA | Uric acid | Continuous | 55-778 |
| 42 | hs_cTnI | Highly sensitive cardiac troponin I | Continuous | 0.001-0.744 |
| 44 | TC | Total cholesterol | Continuous | 1.49-9.12 |
| 45 | TG | Triglyceride | Continuous | 0.45-10.45 |
| 46 | HDL | High density lipoprotein | Continuous | 0.35-3.85 |
| 47 | LDL | Low density lipoprotein | Continuous | 0.28-6.45 |
| 48 | VLDL | Very low-density lipoprotein | Continuous | 3-5122 |
| 49 | Lipoprotein_a | Lipoprotein a | Continuous | 0.1-1116.8 |
| 50 | APOA/APOB | Ratio of apolipoprotein A to apolipoprotein B | Continuous | 0.17-22.40 |
| 51 | Homocysteine | Homocysteine | Continuous | 1.1-82.6 |
| 52 | BG_second | Blood glucose the next day | Continuous | 3.46-17.58 |
| 53 | AFP | Alpha fetoprotein | Continuous | 0.60-12.90 |
| 54 | CEA | Carcinoembryonic antigen | Continuous | 0.31-32.28 |
| 55 | CA125 | CA125 | Continuous | 0.57-448.00 |
| 56 | CA153 | CA153 | Continuous | 1.34-255.75 |
| 57 | CA199 | CA199 | Continuous | 0.60-89.70 |
| 58 | CA724 | CA724 | Continuous | 0.49-70.81 |
| 59 | NSE | Neuron-specific enolase | Continuous | 1.18-85.24 |
| 60 | CRFRA21-1 | CRFRA21-1 | Continuous | 0.94-21.25 |
| 61 | ProGRP | Gastrin-releasing peptide precursor | Continuous | 1.43-333.34 |
| 62 | FT3 | Free triiodothyronine | Continuous | 1.74-16.61 |
| 63 | FT4 | Free tetraiodothyronine | Continuous | 1.39-29.47 |
| 64 | TSH | Thyroid stimulating hormone | Continuous | 0.01-244.00 |
| **SN** | **Predictor** | **Description** | **Types** | **Values** |
| 65 | Folate | Folate | Continuous | 1.80-115.18 |
| 66 | B12 | Vitamin B12 | Continuous | 49.06-1504.00 |
| 67 | AC | Acceleration capacity | Continuous | -14.3882~-1.4272 |
| 68 | DC | Deceleration capacity | Continuous | 1.3475-12.3408 |
| 69 | SDNN | the standard deviation of normal R-R intervals | Continuous | 14-978 |
| 70 | SDANN | the standard deviation of average normal to normal RR intervals | Continuous | 22-214 |
| 71 | RMSSD | The root mean square of successful R-R difference | Continuous | 8-213 |
| 72 | PNN50 | the percentage of successful normal sinus RR intervals with absolute changes exceeding 50ms | Continuous | 0-83 |
| 73 | VLF | Very Low Frequency‌ | Continuous | 20.8-21150.5 |
| 74 | LF | Low Frequency‌ | Continuous | 1.5-5151.1 |
| 75 | HF | High Frequency‌ | Continuous | 1.01-3835.3 |
| 76 | LH/HF | The ratio of the absolute value of LH to HF | Continuous | 0.1208-7.439 |
| 77 | DR | DR = DC +AC | Continuous | -6.18-0.92 |
| 78 | NIHSS_five | the National Institute of Health Stroke Scale at fifth day | Continuous | 0-28 |

***large-artery atherosclerosis，LAA**

***cardioembolism，CE**

***small-artery occlusion lacunar，SAA**

***stroke of other determined etiology，SOE**

***stroke of undetermined etiology，SUE**
