# Supplementary material for: Integrating phase-rectified signal averaging with machine learning to predict stroke-associated infections: a retrospective cohort study
Source: Front Neurol. 2026 Jan 13;16:1653947. doi: 10.3389/fneur.2025.1653947 (PMC12834720; doi:10.3389/fneur.2025.1653947)
Supplement: Supplementary file 2 [file Table_2.docx]

**Supplementary Table S2** SMOTE-processed training set variable features

| Variables | Total (n = 468) | NSAI (n = 234) | SAI (n = 234) | Statistic | *P* |
| --- | --- | --- | --- | --- | --- |
|  |  |  |  |  |  |
| Age, Mean ± SD | 65.72 ± 12.57 | 59.01 ± 11.59 | 72.43 ± 9.59 | t=-13.65 | **<.001** |
| DR, M (Q₁, Q₃) | -0.19 (-0.53, 0.01) | -0.29 (-0.59, -0.10) | -0.08 (-0.40, 0.06) | Z=-5.64 | **<.001** |
| HF, M (Q₁, Q₃) | 85.35 (36.76, 165.05) | 119.50 (67.92, 187.20) | 46.82 (30.12, 113.08) | Z=-7.09 | **<.001** |
| LF, M (Q₁, Q₃) | 239.00 (118.38, 482.75) | 378.40 (200.70, 626.00) | 140.16 (77.14, 327.04) | Z=-8.70 | **<.001** |
| VLF, M (Q₁, Q₃) | 1161.90 (561.69, 2107.43) | 1721.80 (1102.82, 2654.00) | 605.20 (437.54, 1262.73) | Z=-10.98 | **<.001** |
| RMSSD, M (Q₁, Q₃) | 29.23 (21.00, 47.00) | 28.00 (21.00, 44.00) | 30.72 (21.13, 51.18) | Z=-1.63 | 0.103 |
| SDANN, M (Q₁, Q₃) | 68.34 (58.68, 86.00) | 79.00 (64.25, 101.00) | 62.00 (50.03, 72.00) | Z=-10.04 | **<.001** |
| SDNN, M (Q₁, Q₃) | 86.21 (71.37, 112.00) | 103.00 (83.00, 124.75) | 75.80 (67.25, 89.87) | Z=-9.63 | **<.001** |
| DC, M (Q₁, Q₃) | 5.42 (3.83, 6.98) | 6.84 (5.57, 8.00) | 4.16 (3.12, 5.29) | Z=-12.03 | **<.001** |
| B12, M (Q₁, Q₃) | 281.82 (191.28, 368.53) | 284.38 (192.04, 382.31) | 279.38 (191.90, 336.10) | Z=-0.83 | 0.404 |
| FT3, M (Q₁, Q₃) | 3.95 (3.52, 4.57) | 4.19 (3.79, 4.74) | 3.75 (3.40, 4.20) | Z=-7.10 | **<.001** |
| CRP, M (Q₁, Q₃) | 1.44 (0.54, 4.10) | 0.93 (0.50, 2.98) | 1.80 (0.91, 5.97) | Z=-5.95 | **<.001** |
| NIHSS_add, M (Q₁, Q₃) | 4.00 (2.00, 6.00) | 2.00 (1.00, 4.00) | 4.34 (3.39, 8.06) | Z=-9.34 | **<.001** |
| CA125, M (Q₁, Q₃) | 9.89 (6.97, 12.65) | 9.04 (6.83, 11.99) | 10.41 (7.25, 13.80) | Z=-2.64 | **0.008** |
| NG, n(%) |  |  |  | χ²=109.35 | **<.001** |
| No | 361 (77.14) | 228 (97.44) | 133 (56.84) |  |  |
| Yes | 107 (22.86) | 6 (2.56) | 101 (43.16) |  |  |
| Bleeding, n(%) |  |  |  | χ²=11.22 | **<.001** |
| No | 447 (95.51) | 231 (98.72) | 216 (92.31) |  |  |
| Yes | 21 (4.49) | 3 (1.28) | 18 (7.69) |  |  |
| t: t-test, Z: Mann-Whitney test, χ²: Chi-square test | | | | | |
| SD: standard deviation, M: Median, Q₁: 1st Quartile, Q₃: 3st Quartile | | | | | |
