# Supplementary material for: Integrating phase-rectified signal averaging with machine learning to predict stroke-associated infections: a retrospective cohort study
Source: Front Neurol. 2026 Jan 13;16:1653947. doi: 10.3389/fneur.2025.1653947 (PMC12834720; doi:10.3389/fneur.2025.1653947)
Supplement: Supplementary file 3 [file Table_3.docx]

| Variables | Total (n = 118) | NSAI (n = 101) | SAI (n = 17) | Statistic | *P* |
| --- | --- | --- | --- | --- | --- |
|  |  |  |  |  |  |
| Age, Mean ± SD | 59.43 ± 12.19 | 58.36 ± 10.90 | 65.82 ± 17.14 | t=-1.74 | 0.099 |
| DR, M (Q₁, Q₃) | -0.29 (-0.59, -0.07) | -0.33 (-0.68, -0.10) | -0.05 (-0.22, 0.06) | Z=-2.67 | **0.008** |
| HF, M (Q₁, Q₃) | 109.50 (58.07, 214.65) | 115.20 (65.30, 214.50) | 52.40 (25.50, 234.70) | Z=-1.61 | 0.108 |
| LF, M (Q₁, Q₃) | 328.45 (211.05, 554.60) | 354.90 (237.90, 555.00) | 162.50 (85.50, 388.80) | Z=-2.28 | **0.023** |
| VLF, M (Q₁, Q₃) | 1706.55 (1030.40, 2665.15) | 1791.90 (1242.40, 2708.00) | 879.40 (355.80, 1707.80) | Z=-2.86 | **0.004** |
| RMSSD, M (Q₁, Q₃) | 29.00 (22.00, 47.75) | 29.00 (23.00, 47.00) | 22.00 (16.00, 49.00) | Z=-1.16 | 0.245 |
| SDANN, M (Q₁, Q₃) | 82.00 (68.00, 101.00) | 84.00 (70.00, 103.00) | 76.00 (48.00, 81.00) | Z=-2.73 | **0.006** |
| SDNN, M (Q₁, Q₃) | 103.00 (87.25, 127.75) | 106.00 (92.00, 128.00) | 75.00 (58.00, 118.00) | Z=-2.21 | **0.027** |
| DC, M (Q₁, Q₃) | 6.37 (4.62, 7.82) | 6.97 (5.28, 7.94) | 4.14 (2.66, 5.13) | Z=-3.92 | **<.001** |
| B12, M (Q₁, Q₃) | 242.11 (167.32, 334.93) | 245.78 (180.21, 337.56) | 148.63 (105.01, 212.76) | Z=-2.72 | **0.007** |
| FT3, M (Q₁, Q₃) | 4.07 (3.67, 4.46) | 4.17 (3.81, 4.53) | 3.63 (2.84, 3.90) | Z=-3.73 | **<.001** |
| CRP, M (Q₁, Q₃) | 1.27 (0.50, 2.91) | 1.25 (0.50, 2.63) | 1.82 (0.50, 4.59) | Z=-0.87 | 0.382 |
| NIHSS add, M (Q₁, Q₃) | 3.00 (1.00, 5.00) | 2.00 (1.00, 5.00) | 6.00 (3.00, 12.00) | Z=-3.27 | **0.001** |
| CA125, M (Q₁, Q₃) | 9.21 (6.44, 13.28) | 8.28 (6.41, 12.62) | 14.27 (7.54, 18.75) | Z=-2.07 | **0.038** |
| NG, n(%) |  |  |  | χ²=50.94 | **<.001** |
| No | 107 (90.68) | 100 (99.01) | 7 (41.18) |  |  |
| Yes | 11 (9.32) | 1 (0.99) | 10 (58.82) |  |  |
| Bleeding, n(%) |  |  |  | - | 0.054 |
| No | 115 (97.46) | 100 (99.01) | 15 (88.24) |  |  |
| Yes | 3 (2.54) | 1 (0.99) | 2 (11.76) |  |  |
| t: t-test, Z: Mann-Whitney test, χ²: Chi-square test, -: Fisher exact | | | | | |
| SD: standard deviation, M: Median, Q₁: 1st Quartile, Q₃: 3st Quartile | | | | | |

**Supplementary Table S3** Features of Each Variable in the Validation Set.
