# Supplementary material for: Integrating phase-rectified signal averaging with machine learning to predict stroke-associated infections: a retrospective cohort study
Source: Front Neurol. 2026 Jan 13;16:1653947. doi: 10.3389/fneur.2025.1653947 (PMC12834720; doi:10.3389/fneur.2025.1653947)
Supplement: Supplementary file 4 [file Data_Sheet_1.pdf]

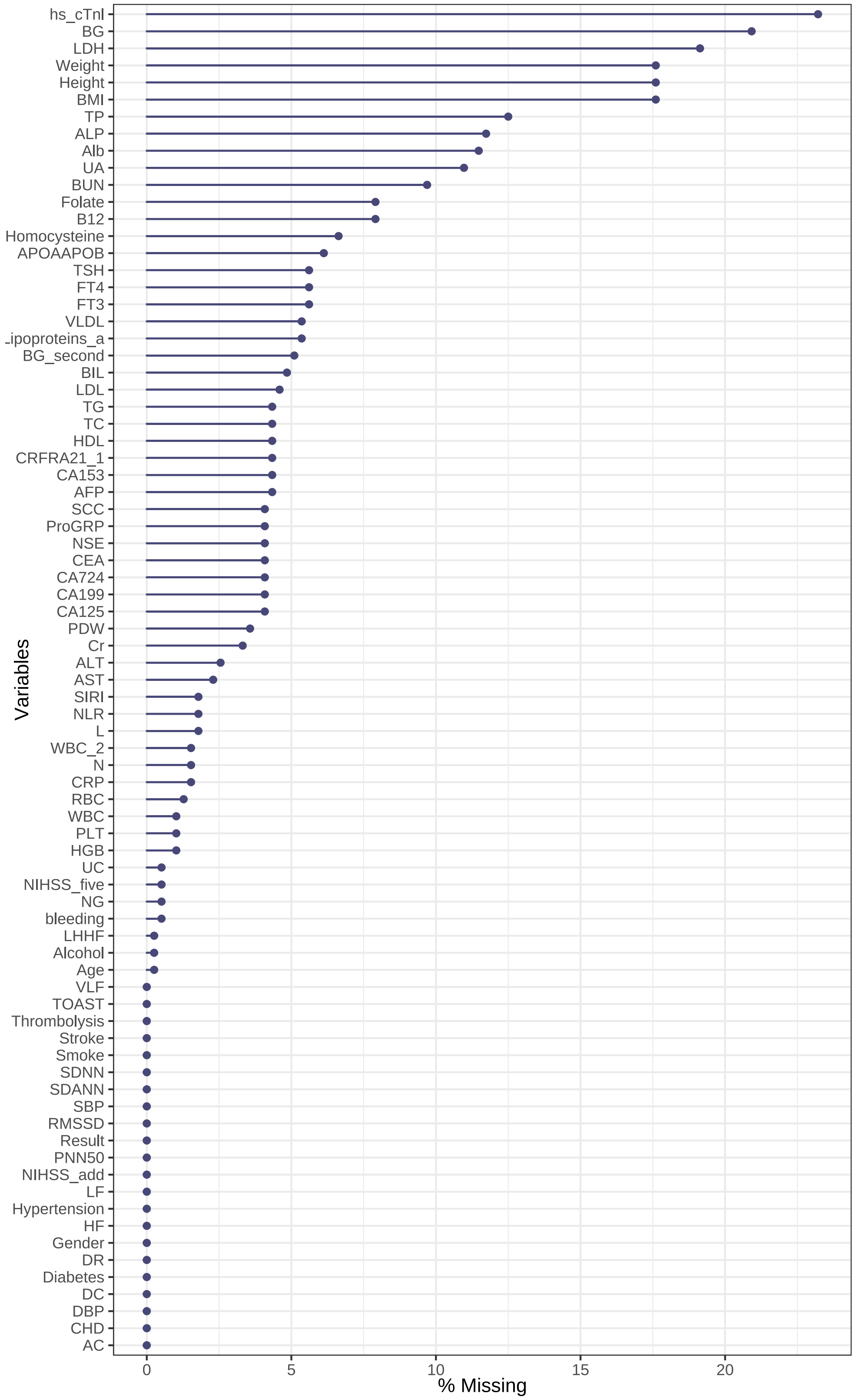

Figure S1. The specifics of the missing values are shown, Variables with more than 20% missing data were excluded from the analysis. Missing rates for BG and hs-cTnl are greater than 20%. The full names and abbreviations of the included features are listed in Supplementary Table S1.
